# Multi‐Omics Analysis of Aberrances and Functional Implications of IRF5 in Digestive Tract Tumours

**DOI:** 10.1111/jcmm.70433

**Published:** 2025-02-24

**Authors:** Long Yao, Xiu Chen, Yanxin Fang, Yunlong Huang, Kaiming Wu, Xin Huang, Junrui Xu, Renquan Zhang

**Affiliations:** ^1^ Department of Thoracic Surgery The First Affiliated Hospital of Anhui Medical University Hefei Anhui China; ^2^ Department of Cardiothoracic Surgery Anhui No. 2 Provincial People's Hospital Hefei Anhui China

**Keywords:** gene‐pair signature, oesophageal cancer, senescence, single‐cell, spatial transcriptomics

## Abstract

Oesophageal cancer (EC) is a common gastrointestinal malignancy and includes oesophageal squamous cell carcinoma (ESCC) and oesophageal adenocarcinoma (EAC) sub‐types. Gene signatures predicting patient outcomes are not routinely used in clinical practice, particularly owing to batch effects and data standardisation. Here, we sought to establish and validate a reliable signature of senescence‐related genes (SRGs) that would aid in predicting prognosis in patients with EC. We downloaded transcriptomics data, and a novel pairwise comparison algorithm selected valid SRG pairs (SRGPs) to construct a prognostic SRGP signature. The SRGPs were verified using Kaplan–Meier survival and receiver operating characteristic curve analyses. Additionally, the relationships between the SRGP signatures and prognosis, immune cell infiltration and chemotherapeutic drug responsiveness were evaluated. The random forest algorithm identified the most clinically significant genes, followed by experimental validation. 19 and 26 SRGP signatures were created for ESCC (*n* = 81) and EAC (*n* = 79), respectively. Patients with EC were divided into two groups based on the median risk score. The Kaplan–Meier analysis demonstrated significant differences in overall survival between the ESCC and EAC groups (*p* < 0.001). The sub‐types exhibited different immune signatures. *IRF5* was the most clinically significant gene for ESCC. It was highly expressed in ESCC cells, and *IRF5* knockdown inhibited cell migration and proliferation, while promoting apoptosis and senescence. The SRGP signature may predict prognosis and immunotherapeutic responses, and *IRF5* is a potential target gene for ESCC.

**Abbreviations**
CI: confidence interval
Coeff: coefficient
EAC: oesophageal adenocarcinoma
EC: oesophageal cancer
EdU: 5‐ethynyl‐2′‐deoxyuridine
ESCC: oesophageal squamous cell carcinoma
GDSC: Genomics of Drug Sensitivity in Cancer
GSVA: gene set variation analysis
HR: hazard ratio
IC50: half‐maximum inhibitory concentration
ICI: immune checkpoint inhibitor
KEGG: Kyoto Encyclopedia of Genes and Genomes
K–M: Kaplan–Meier
LASSO: least absolute shrinkage and selection operator
OS: overall survival
ROC: receiver operating characteristic
RSF: random survival forest
SRG: senescence‐related gene
SRGP: senescence‐related gene pair
TCGA: The Cancer Genome Atlas
TME: tumour microenvironment
WB: western blot

## 1 Introduction

Oesophageal cancer (EC) is one of the most common gastrointestinal cancers worldwide. It is the second most prevalent solid tumour after lung cancer, with a high incidence and mortality rate in China [1]. The incidence and histopathology of EC vary across geographic regions, and EC imposes high societal burdens, including in East Asia [2]. Oesophageal squamous cell carcinoma (ESCC) and oesophageal adenocarcinoma (EAC) are the most common EC types [3]. ESCC is a malignancy of squamous epithelial cells characterised by the over‐expression of epidermal growth factor receptor and TP53 [4]. EAC is a primary malignant columnar epithelial tumour. The poor prognosis of EC is primarily attributed to late diagnosis, frequent metastases and treatment resistance [5]. EC treatments have evolved significantly owing to multidisciplinary approaches. However, patients with late‐stage EC have a short survival duration [6]. Thus, there is an urgent need to develop new diagnostic, therapeutic and prognostic strategies to improve the overall survival (OS) of patients with EC.

Senescence marks the permanent arrest of the cell cycle and progressive functional decline that may lead to tissue dysfunction [7]. It is a risk factor for several diseases, including heart disease [8], diabetes [9] and malignant tumours [10]. Senescence‐related genes (SRGs) are involved in regulating tumour‐cell senescence. These genes inhibit tumour growth by regulating cell senescence, but they may also promote invasion and metastasis. Senescence is a strong prognostic indicator of decreased survival in various cancers [11, 12, 13]. Considering the important role of senescence, here, we aimed to generate an SRG signature that can differentiate between EC risk groups to help select individual treatments.

With the rapid development of bioinformatic tools, cancer can be readily studied in detail. Several studies have identified gene signatures predicting outcomes in patients with EC [14, 15]. However, these signatures are not commonly used in clinical practice owing to various factors, including batch effects and data standardisation. Nevertheless, researchers have developed a pairwise comparison method [16] to overcome these problems. Many predictions made using this algorithm are robust [17, 18].

The aim of this study was to establish a prognostic signature of SRG pairs (SRGPs) through a pairwise comparison approach. To the best of our knowledge, this is the first bioinformatic study to identify key SRGs in EC and the extent of immune cell infiltration. These genes further our understanding of the molecular mechanisms underlying EC and provide evidence for early detection, early diagnosis and early treatment of the disease.

## 2 Materials and Methods

### 2.1 Identification of IRF5 Spatial Phenomenon Through GI Spatial Transcriptome

The spatial transcriptomics data of pan‐digestive tract tumour patients were first downloaded through the 10X database as well as the GSE database (GSE203612), followed by deconvolution of the spatial transcriptomics data. We explored cellular localisation, calculation of per spot maxima in cellular localisation as well as single‐gene spatial transcriptomics localisation, and Spearman's correlation of gene expression with microenvironmental components to validate the importance of IRF5 at the spatial level.

### 2.2 Pan‐Digestive Tract Tumour Expression

We next validated IRF5 expression through TCGA pan‐GI data. We analysed the differences in IRF5 expression in the TCGA pan‐GI tract as well as in the combined GTEx database. Subsequently, we analysed the expression of IRF5 in the pan‐digestive tract tumour single‐cell dataset and also analysed the expression differences of IRF5 in copy number variants in the pan‐digestive tract data. Finally, IRF5 was analysed by Spearman correlation to correlate with multiple algorithmic immune infiltration.

### 2.3 Data Acquisition

We downloaded gene expression data and the corresponding clinical data for EC from The Cancer Genome Atlas (TCGA; https://tcga‐data.nci.nih.gov/tcga/) database. The oesophageal squamous carcinoma dataset comprised data of 11 normal and 70 squamous carcinoma samples, whereas the adenocarcinoma dataset contained data of 8 normal and 71 adenocarcinoma samples. Genes with mean expression values below 1 and samples from patients for whom survival information was unavailable were removed from analysis. Ultimately, data of the remaining 81 patients with ESCC and 79 patients with EAC were included in the study. SRGs were retrieved from the CellAge database.

### 2.4 Identification of SRGPs

We downloaded transcriptomic data from the TCGA database and acquired SRGs from the Database of Cell Senescence Genes (CellAge; https://genomics.senescence.info/cells/). The intersection of these datasets was used to identify SRGs with clinical information. In this study, we proposed an algorithm based on the comparison of gene‐pair expression levels. Firstly, we selected two genes from a senescence gene list to form a pair, which is called the SRGPs. For each SRGP, we compared the expression levels of its two genes (referred to as the first SRG and the second SRG).

Calculation of gene‐pair expression levels:

- If the expression level of the first SRG is higher than that of the second SRG, the expression level of the SRGP is defined as 1.
- If the expression level of the first SRG is lower than that of the second SRG, the expression level of the SRGP is defined as 0.

Gene‐pair screening criteria:

- We calculated the expression level distribution of each SRGP in all samples.
- If the expression level of a certain SRGP is 0 or 1 in more than 80% of the samples, it is considered that it is difficult to provide survival discriminatory information. Therefore, it will be removed to prevent the incorporation of noisy features.

### 2.5 Establishment and Evaluation of Signatures

The univariate Cox regression and least absolute shrinkage and selection operator (LASSO) analyses were performed to identify significant SRGPs associated with OS of patients with EC using the glmnet and survival packages. We separately performed a univariate Cox analysis of ESCC and EAC. The univariate Cox analysis comprises nested linear equations and creates a new linear regression equation by exponential transformation. The hazard ratio (HR) directly demonstrates the relative magnitude of the rate of death. The event of interest was used to explore a non‐linear effect of genes on the risk of death. Results with a *p* < 0.05 determined using the univariate Cox analysis were considered statistically significant. Subsequently, the LASSO analysis was used to choose statistically significant SRGPs to establish prognostic signatures. In terms of the model establishment, we chose the Lasso‐cox model. This is because the linear model is not only simple and intuitive but also easy to interpret, and it can clearly reveal the specific contribution of each gene pair to prognosis. The data cleaning steps included removing missing values and non‐tumour samples to ensure the integrity and accuracy of the data. Meanwhile, we converted the survival time from days to years to standardise the time unit. The Lasso regression was implemented through the glmnet package. We specified the family parameter as “cox” and set the alpha parameter to 1. Then we used the cv.glmnet function to perform 10‐fold cross‐validation to select the optimal lambda value. From the training results, we extracted the model coefficients and feature names corresponding to the optimal lambda value and screened out the non‐zero coefficients and their corresponding gene‐pair names. The final prognostic risk score model was established by multiplying *β* (Coeff) values with the gene. The risk score derived from the signature was calculated using the following formula:

$$\text{Risk}\;\text{score}=\sum_{i=1}^{n}{\text{Coeff}}_{i}\times \text{gene}\;{\text{pair}}_{i}$$

Subsequently, patients were assigned risk scores and divided into high‐ and low‐risk groups based on the median of the risk score. Kaplan–Meier (K–M) analysis was used to compare the OS of the risk groups. We utilised receiver operating characteristic (ROC) curves to evaluate prognostic performance, and time‐dependent ROC curves were obtained using the inverse probability censoring weighted method. The survival risk curve and scatter plot demonstrated the prognostic states of the patients. Finally, we examined the relationships between risk score and the following crucial clinicopathological parameters: tumour size, lymph node and stage, describing relative risks by HR and the 95% confidence interval (CI).

### 2.6 Gene Set Variation Analysis (GSVA)

We performed GSVA using R software to assess differences between pathway and hallmark gene sets between the risk groups. A threshold false discovery rate value of < 0.05 was used to determine statistical significance.

### 2.7 Evaluation of Immune Cell Infiltration and Prediction of Drug Responses

We used the ESTIMATE R package to estimate the proportions of immune‐stromal components in the tumour microenvironment (TME) [19]. We used the TIMER2.0 database [20] (http://timer.comp‐genomics.org/) to assess immune cell infiltration in ESCC and EAC. Several algorithms were used to calculate immune cell infiltrates. A bubble diagram was used to illustrate the correlation. We predicted the responses of patients to therapeutic drugs according to data from the Genomics of Drug Sensitivity in Cancer (GDSC) database (https://www.cancerrxgene.org). The half‐maximum inhibitory concentration (IC_50_) was calculated using the R software package pRRophic.

### 2.8 Identification of Hub Genes

We used the random survival forest (RSF) package to identify genes with relative importance values > 0.5. These genes were selected for further experimental validation.

### 2.9 Cell Culture and Transfection

Human ESCC cell lines (KYSE150, KYSE180, KYSE410 and KYSE450) and the human embryonic oesophageal cell line (SHEE) were purchased from iCell Bioscience Inc. (Shanghai, China). The cells were cultured in RPMI‐1640 medium (Wisent, Canada) supplemented with 10% fetal bovine serum (Wisent, Canada) and maintained at 37°C under 5% CO_2_. Si‐IRF5 and negative control siRNA were purchased from Tsingke (Beijing, China). Lipofectamine 2000 (Gibco, UK) was used for all transfections. Western blot (WB) analysis was used to assess the silencing efficiency.

### 2.10 WB Analysis

Briefly, cell lysates were prepared using RIPA buffer (# P0013B, Beyotime, Shanghai, China) and protein concentration was determined using the Bradford Protein Assay Kit (#P0399S, Beyotime, Shanghai, China). Twenty micrograms of protein was resolved by performing sodium dodecyl sulfate‐polyacrylamide gel electrophoresis using 10% resolving gels and electrotransferred onto a nitrocellulose membrane(#HATF00010, Millipore Corp, Billerica, MA, USA). The membrane was blocked with 5% skimmed milk for 1 h at room temperature (20°C) and then with a primary antibody (IRF‐5, 1:1000, 10547‐1‐AP; Proteintech, Chicago, USA) at 4°C overnight. The membrane was then incubated with a secondary antibody (Goat polyclonal anti‐mouse IgG, 1:10,000, Cat# 511103; Zen Bioscience, Chengdu, China) for 1 h at room temperature.

### 2.11 Wound Healing Assay

The cells were plated in a six‐well plate and transfected. Then, a scratch or a wound is carefully created in the cell monolayer using a sterile pipette tip or a specialised tool when the cell density reached 90%. This creates a cell‐free area or gap in the middle of the confluent cell layer. After that, the cells are washed gently to remove debris and floating cells, and then fresh culture medium is added. The cells are then incubated at 37°C under 5% CO_2_. Over time, the cells at the edges of the wound start to migrate and proliferate towards the center of the wound area. The process of the wound closing can be monitored and photographed at different time points (at 0 and 24 h). The wound healing area was analysed using ImageJ software (National Institutes of Health, Bethesda, MD, USA).

### 2.12 Colony Formation

First, cells are seeded at a proper density (1000 cells/well) into culture dishes containing suitable growth medium. Then, the cells are incubated at 37°C under 5% CO_2_. Over 14 days, the cells divide and form colonies. Each colony represents a group of daughter cells that originated from a single cell. Finally, the colonies are fixed and stained with 0.2% crystal violet (#C0121, Beyotime, Shanghai, China) for 30 min at room temperature, washed with sterile water and photographed. The number and size of the colonies can be counted and analysed.

### 2.13 5‐Ethynyl‐2′‐Deoxyuridine (EdU) Assay

Briefly, the cells were plated in a six‐well plate and transfected. EdU (# C0078S; Beyotime, Shanghai, China) was diluted to 20 μM with complete culture medium. 1000 μL of the diluted EdU working solution was added into each well so that the final concentration is 10 μM. Then, it was continued to incubate at 37°C for 2 h. The cell culture medium was removed and washed with phosphate buffered saline (PBS) for 1–2 times, 3 min each time. 50 μL of 4% paraformaldehyde was added into each well to fix the cells at room temperature for 30 min. The fixative was removed and washed with PBS for 3 times, 3–5 min each time. The PBS was removed, and 100 μL of permeabilisation solution (PBS containing 0.5% Triton X‐100) was added into each well and incubated at room temperature for 10–15 min. The permeabilisation solution was removed and washed with PBS for 1–2 times, 3–5 min each time. The reaction solution was prepared according to the instructions of the kit. 1 mL of the reaction solution was added into each well, and the culture plate was gently shaken to ensure that the reaction solution evenly covers the samples. It was incubated at room temperature in the dark for 30 min. The reaction solution was removed and washed with PBS for 3 times, 3–5 min each time. The DAPI reaction solution was diluted to 1× with deionised water. After removing the PBS washing solution, DAPI was added into each well and stained at room temperature in the dark for 10 min. DAPI was removed and washed with PBS for 3 times, 3–5 min each time. The cells were photographed under a fluorescence microscope (DFC7000T; Leica, German).

### 2.14 Apoptosis Assay

The cells were plated in a six‐well plate, transfected and cultured for 24 h. An apoptosis assay kit (#C1062M, Beyotime, China) was used to measure apoptosis. The cell culture medium was aspirated into a suitable centrifuge tube. The adherent cells were washed once with PBS, and then an appropriate amount of trypsin cell digestion solution was added to digest the cells. It was incubated at room temperature until the adherent cells could be detached by gentle pipetting. Then the trypsin cell digestion solution was aspirated. It is necessary to avoid excessive digestion by trypsin. The cell culture medium collected in the previous step was added, and the cells were gently pipetted to detach them, transferred into the centrifuge tube and centrifuged at 1000 *g* for 5 min, discard the supernatant, collect the cells, gently resuspend the cells with PBS, and count them. Take 50,000–100,000 resuspended cells, centrifuge at 1000 *g* for 5 min, discard the supernatant, add 195 μL of Annexin V‐FITC binding solution to gently resuspend the cells. Add 5 μL of Annexin V‐FITC and mix gently. Add 10 μL of propidium iodide staining solution and mix gently. Incubate at room temperature (20°C–25°C) in the dark for 10–20 min and then place it in an ice bath. Aluminium foil can be used for light shielding. Apoptotic cells were analysed using a CytoFLEX Cytometer (Beckman coulter, USA).

### 2.15 Cellular Senescence Assay

The cells were plated in a six‐well plate, transfected and cultured for 24 h. The cells were washed and incubated using the Senescence β‐Galactosidase Staining Kit (#C0602, Beyotime, Shanghai, China). For the cells cultured in a 6‐well plate, the cell culture medium was aspirated and the cells were washed once with PBS. Then, 1 mL of β‐galactosidase staining fixative was added and the cells were fixed at room temperature for 15 min. The cell fixative was aspirated, and the cells were washed with PBS 3 times, 3 min each time. The PBS or HBSS was aspirated, and 1 mL of staining working solution was added into each well. It was incubated at 37°C overnight and observed using a fluorescence microscope (DFC7000T; Leica).

### 2.16 Statistical Analysis

All analyses were performed using R (version 4.2.1; R Foundation for Statistical Computing, Vienna, Austria, https://www.R‐project.org). All analyses were conducted using R (version 4.2.1; R Foundation for Statistical Computing, Vienna, Austria, https://www.R‐project.org) and GraphPad Prism 9.0 (GraphPad Software, La Jolla, CA, USA). Before carrying out the differential analysis, we first used the scale function to convert the gene expression levels into *z*‐scores. The conversion formula is (*x* − *μ*)/*σ*, so as to facilitate subsequent statistical analyses better. To evaluate the association between OS and gene expression, we utilised the survival and survminer packages in R to conduct Kaplan–Meier analysis and univariate Cox regression analysis (implemented through the coxph() function). In the results, we used the HR and 95% CI to describe the relative risk. Meanwhile, in the Kaplan–Meier analysis, we adopted the log‐rank test (implemented through the survdiff() function), with a significance level of *p* < 0.05, to test the significance of differences in survival time. For the comparison of discrete variables between groups (such as tumour versus non‐tumour, binary classification), we adopted the non‐parametric Wilcoxon rank sum test (implemented through the wilcox.test() function). When it was necessary to detect the significance among multiple variables, we then used the Kruskal–Wallis rank sum test. In terms of data visualisation, all box plots were accompanied by violin plots to display the data distribution. Among them, the middle line represents the median, the upper and lower limits of the box represent the third quartile and the first quartile respectively and the whiskers represent 1.5 times the interquartile range. Outliers beyond this range were also shown as part of the box plot. In terms of correlation analysis, for data with a normal distribution or data on the same scale, we adopted the Pearson correlation analysis (implemented through the cor.test(method = ‘pearson’) function); while for data with a non‐normal distribution or data on different scales, we adopted the Spearman correlation analysis (implemented through the cor.test(method = ‘spearman’) function). In addition, we also used the GSVA package to evaluate the activity of pathways related to malignant features and selected the *z*‐score parameter for the evaluation. To evaluate the predictive performance of genes on the model, we used the ‘timeROC’ package to conduct ROC curve analysis, and the censored data adopted the km method. Finally, it should be noted that all statistical tests were two‐sided tests, and a *p*‐value < 0.05 was considered statistically significant, and a *p*‐value < 0.0001 was considered extremely significant. Statistical significance was represented as *p* < 0.05, *p* < 0.01, *p* < 0.001 and ****p < 0.0001 in the results.

## 3 Results

### 3.1 Spatial Multi‐Omics Mapping of IRF5

**FIGURE 1:**
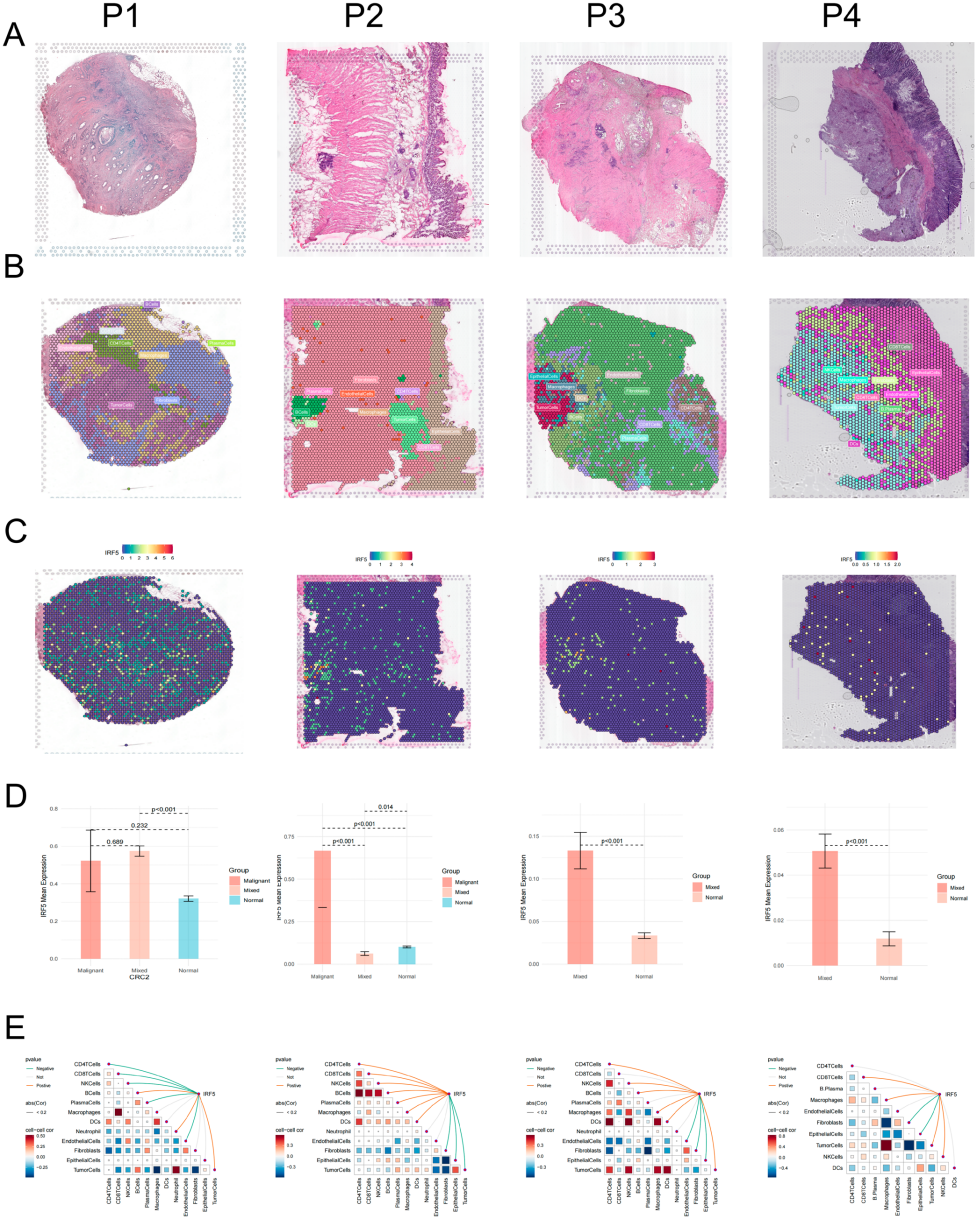
Spatial multi‐omics mapping of IRF5. (A) High‐resolution HE‐stained sections. (B) Cellular components based on the deconvolution algorithm. (C) IRF5 spatial transcriptome localization. (D) Difference in IRF5 expression in the malignant region, mixed malignant region and normal region. (E) The spearman correlation between gene expression and microenvironmental components.

Spatial transcriptomics data were collected from a total of 3 patients with colon cancer, 1 patient with gastric cancer and 1 patient with liver metastasis from colon cancer, and Figure 1A shows the high‐resolution HE‐stained sections of these patients (P1–P3: CRC; P4: STAD; P5: colon cancer liver metastasis). Then, the cellular components (Figure 1B and Figure S1B) as well as the spatial localisation of IRF5 (Figure 1C and Figure S1C) were calculated after spatial transcriptomics deconvolution, and it was found that IRF5 was mainly localised to tumour cells, DC cells and B cells. Meanwhile, IRF5 was highly expressed in malignant regions in the expression difference between colon cancer (Figure 1D (P1‐3)) and gastric cancer (P4); interestingly, in patients with liver metastases from colon cancer (Figure S1D), we found that IRF5 was lowly expressed in malignant regions and highly expressed in mixed malignant regions, which may be related to the presence of spatial variability. Spearman correlation analysis revealed that IRF5 was positively correlated with tumour cells, DC cells, and B cells and negatively correlated with epithelial cells and fibroblasts (Figure 1E).

### 3.2 Expression of IRF5

**FIGURE 2:**
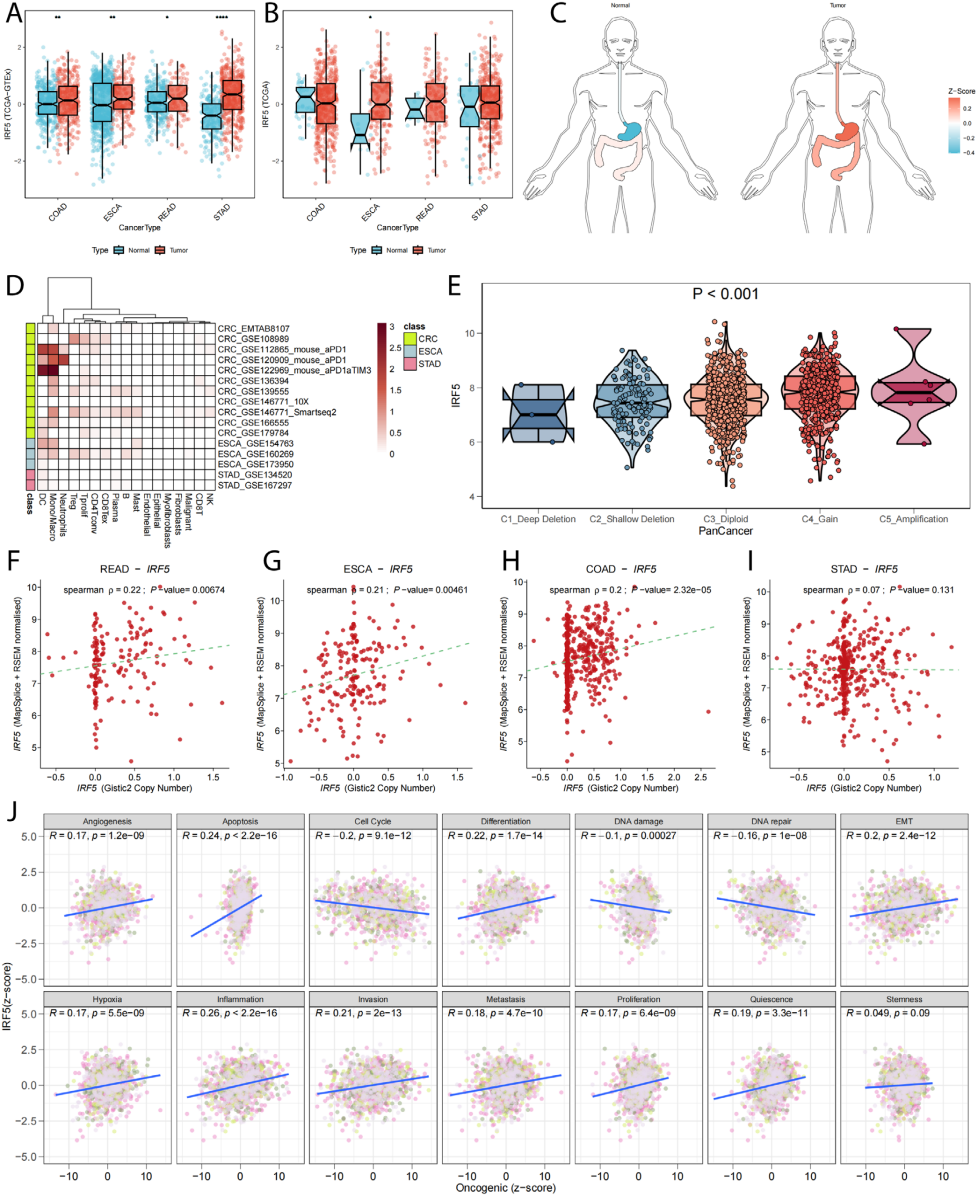
Expression of IRF5. (A) IRF5 was highly expressed in pan‐GI tumours in TCGA combined with GTEx analysis. (B) ESCA expression was dysregulated only in the TCGA dataset. (C) IRF5 expression difference organograms. (D) Single‐cell analysis suggests that IRF5 expression is predominantly in immune cells, and that expression is present in malignant cells as well. (E) Expression differs by the type of copy number variation, and a trend of increased gene expression was observed as the copy number amplification increase the trend of increasing gene expression, (F–I) Spearman correlation analysis was consistent with the previous, as the copy number scoring of gistic2 increased, the gene expression increased. (J) Pearson correlation was assessed between the gene expression and the GSVA scoring of the 14 tumour statuses.

By downloading TCGA pan‐GI data as well as normal samples from GTEx, we found that IRF5 was highly expressed in pan‐GI tumours (Figure 2A); however, when the data were restricted to TCGA only, IRF5 expression was dysregulated only in ESCA (Figure 2B). Expression difference organograms (Figure 2C) were visualised for the above results. Single‐cell data analysis suggested that IRF5 was mainly expressed in DC cells and macrophages, and malignant cells were also expressed (Figure 2D). Copy number variant analysis revealed that expression varied by type (Figure 2E), with increased gene expression with increasing copy number amplification. Spearman correlation analysis revealed that increased gistic2 copy number scoring was followed by increased gene expression (Figure 2F–I). Finally, assessment of gene expression in relation to 14 tumour states revealed that IRF5 function may be associated with anti‐apoptosis, epithelial‐mesenchymal transition (EMT) and inflammatory response (Figure 2J).

### 3.3 Study Populations

We initially obtained 85 ESCC and 80 EAC samples. Patients for whom survival information was not available were excluded from the analysis, resulting in the inclusion of 81 ESCC and 79 EAC samples in the study.

### 3.4 Data Acquisition and SRGP Identification

**FIGURE 3:**
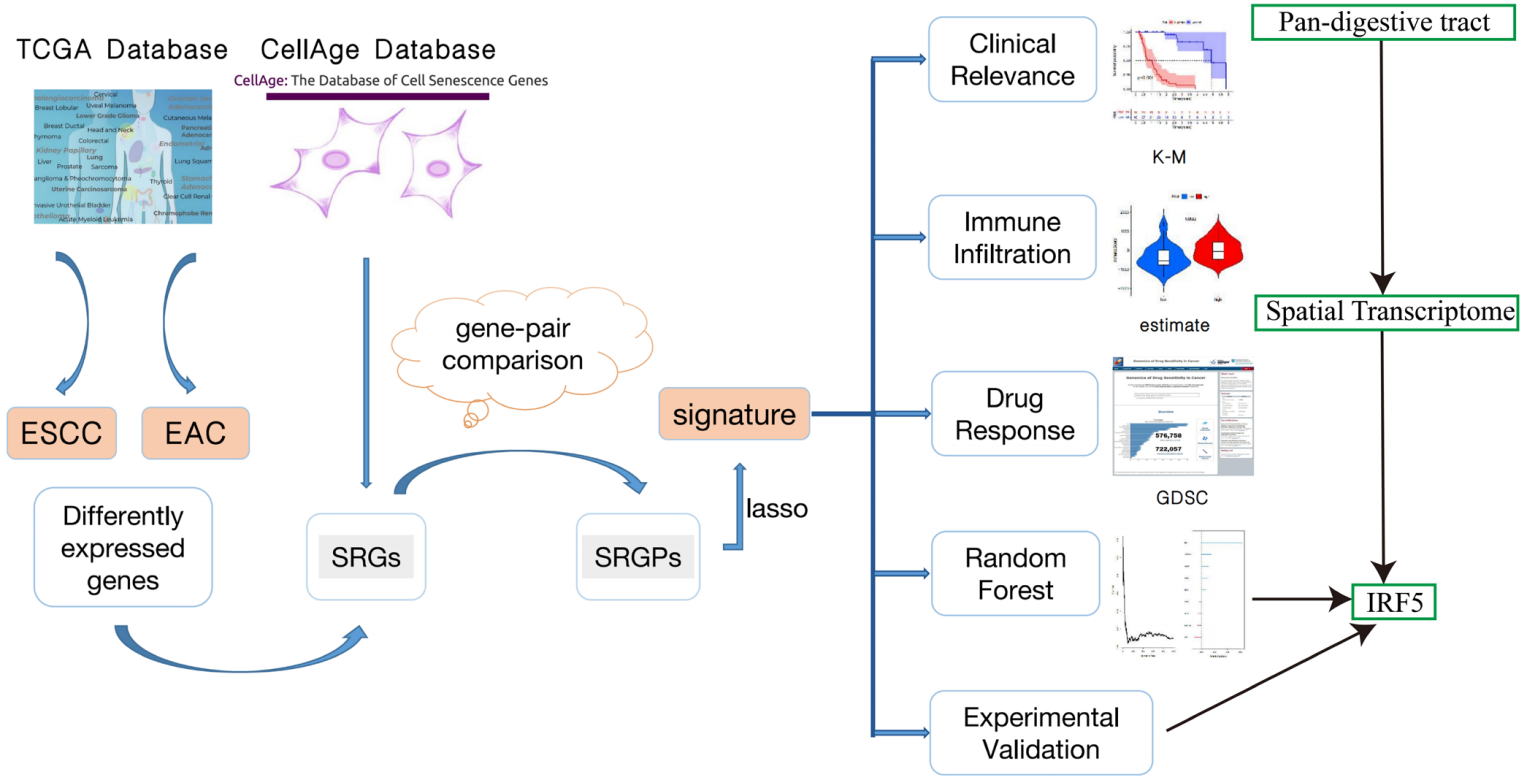
Flowchart of the study.

The study flowchart is shown in Figure 3. Sequencing data and relative clinical data were acquired from TCGA. In total, 279 SRGs were retrieved from the CellAge database. We generated 8160 SRGPs for ESCC and 8501 SRGPs for EAC using the gene‐pair comparison algorithm.

### 3.5 Establishment and Assessment of SRGP Signatures

**FIGURE 4:**
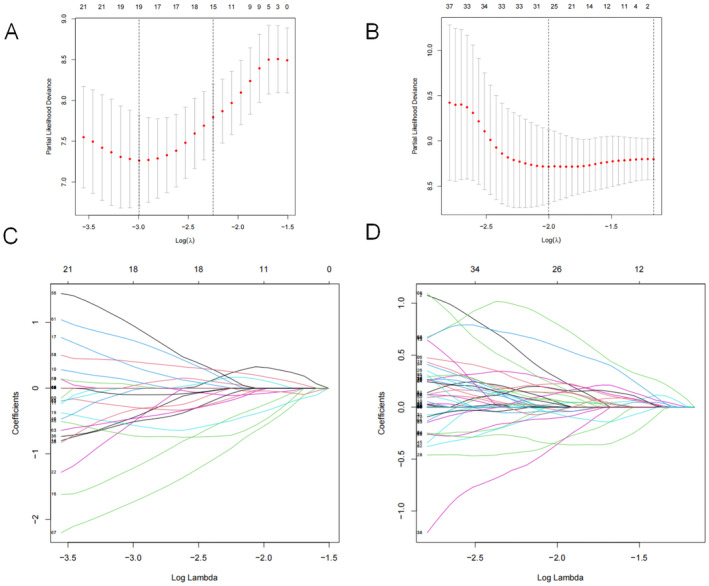
Construction of a risk signature using the least absolute shrinkage and selection operator (LASSO) analysis. Partial likelihood deviances for (A) oesophageal squamous cell carcinoma (ESCC) and (B) oesophageal adenocarcinoma (EAC). Coefficient profiles of senescence‐related gene pairs for (C) ESCC and (D) EAC.

**FIGURE 5:**
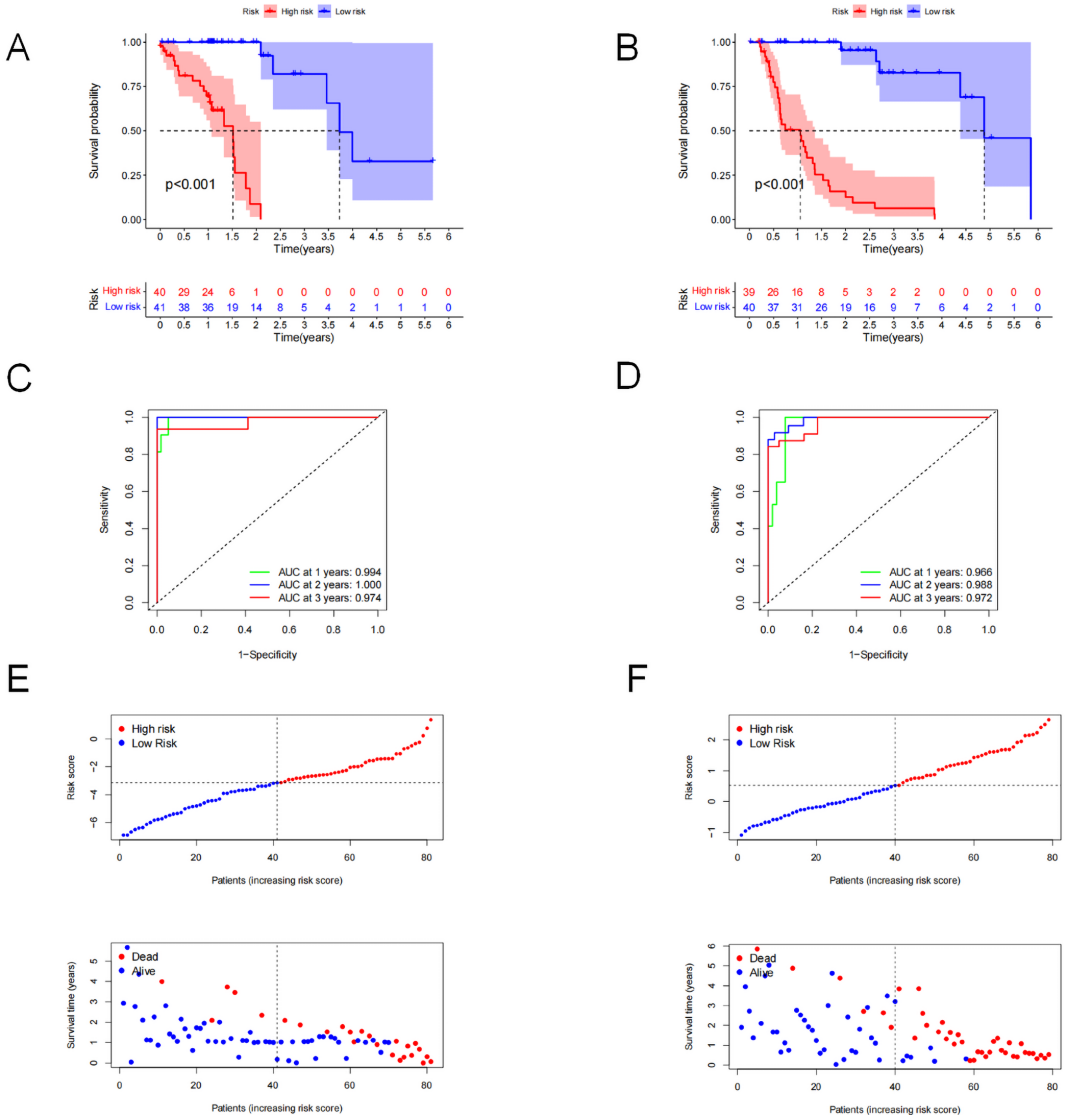
Kaplan–Meier analysis of high‐ and low‐risk patients (red and blue, respectively) with (A) oesophageal squamous cell carcinoma (ESCC) and (B) oesophageal adenocarcinoma (EAC). Receiver operating characteristic curves for (C) ESCC and (D) EAC. Survival risk curves (top) and sand scatter plots (bottom) for (E) ESCC and (F) EAC.

The univariate Cox analysis was used to identify 70 prognostic SRGPs associated with ESCC and 187 with EAC (*p* < 0.01) from the gene pairs. We then performed LASSO analysis (Figure 4) to generate a signature of 19 SRGPs for ESCC (Table S1) and 26 SRGPs for EAC (Table S1). Each patient was assigned a risk score derived from their SRGP signature. We divided the patients into two risk groups based on the median of the risk score in the ESCC and EAC groups. The low‐risk group had a better survival rate among patients with ESCC (Figure 5A) and EAC (Figure 5B) (*p* < 0.001) than the high‐risk group according to the K–M survival analysis. The signatures had excellent sensitivity and specificity for the prediction efficiency, with an area under the ROC curve (AUC) value of > 0.9 and AUCs of 0.974 and 0.972 for the 3‐year prediction between patients with ESCC and EAC, respectively (Figure 5C,D). The survival data are presented in Figure 5E. Similar results were observed for EAC (Figure 5F). Subsequently, a subgroup analysis was performed to validate the predictive value of the signatures for multiple clinicopathological parameters (tumour size, lymph node and tumour stage). The signatures showed good discrimination for EC cases stratified by tumour size (T1–2 or T3–4), lymph node (N0 or N1–3) and tumour stage (I–II or III–IV) (Figure S2).

**FIGURE 6:**
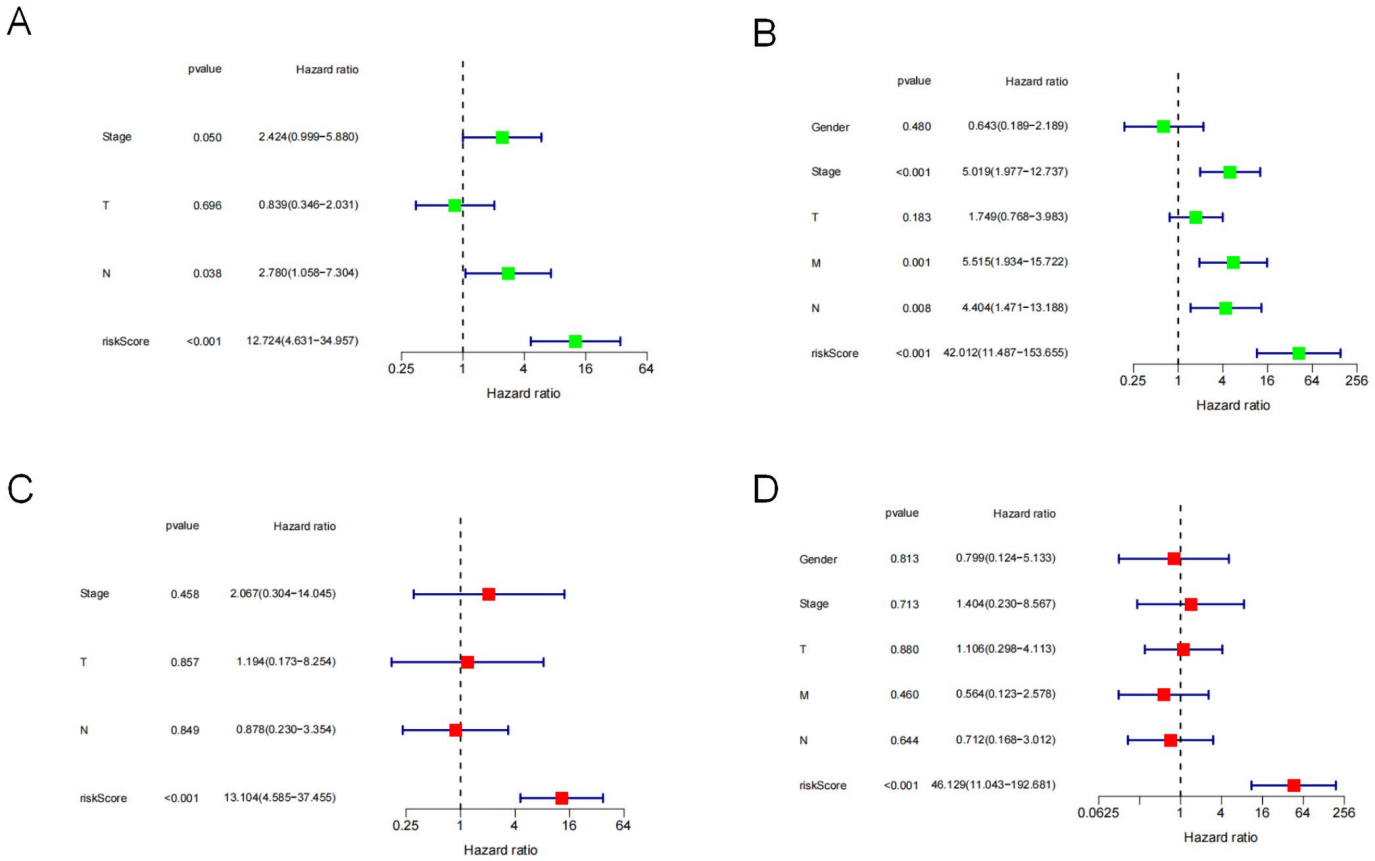
Hazard ratios from (A) univariate and (C) multivariate analyses for oesophageal squamous cell carcinoma. (B) Univariate and (D) multivariate analyses for oesophageal adenocarcinoma.

The univariate Cox analysis indicated that tumour size, lymph node, tumour stage and risk score correlated with OS. The risk score was a strong indicator for ESCC ([HR] = 12.724, 95% CI: 4.631–34.957, *p* < 0.001) (Figure 6A) and EAC (HR = 42.012, 95% CI: 11.487–153.655, *p* < 0.001) (Figure 6B). The multivariate Cox analysis demonstrated that the signature independently predicted ESCC prognosis (Figure 6C). The risk score also independently predicted EAC (Figure 6D).

GSVA was used to assess the differences in biological processes between ESCC and EAC. The Kyoto Encyclopedia of Genes and Genomes (KEGG) pathway analysis demonstrated that the enriched pathways were T‐cell receptor, RIG‐1 like receptor, chemokine and JAK/STAT signalling for ESCC (Figure S3A) and chemokine, T‐cell receptor and B‐cell receptor signalling for EAC (Figure S3B). The HALLMARK analysis illustrated that prognostic gene sets were enriched in HALLMARK_INTERFERON_GAMMA_RESPONSE, HALLMARK_INFLAMMATORY_RESPONSE and HALLMARK_E2F_TARGETS in ESCC (Figure S3C), whereas HALLMARK_MYC_TARGETS_V1, HALLMARK_G2M_CHECKPOINT and HALLMARK_APOPTOSIS had prognostic values in EAC (Figure S3D).

### 3.6 Immune Cell Infiltration

**FIGURE 7:**
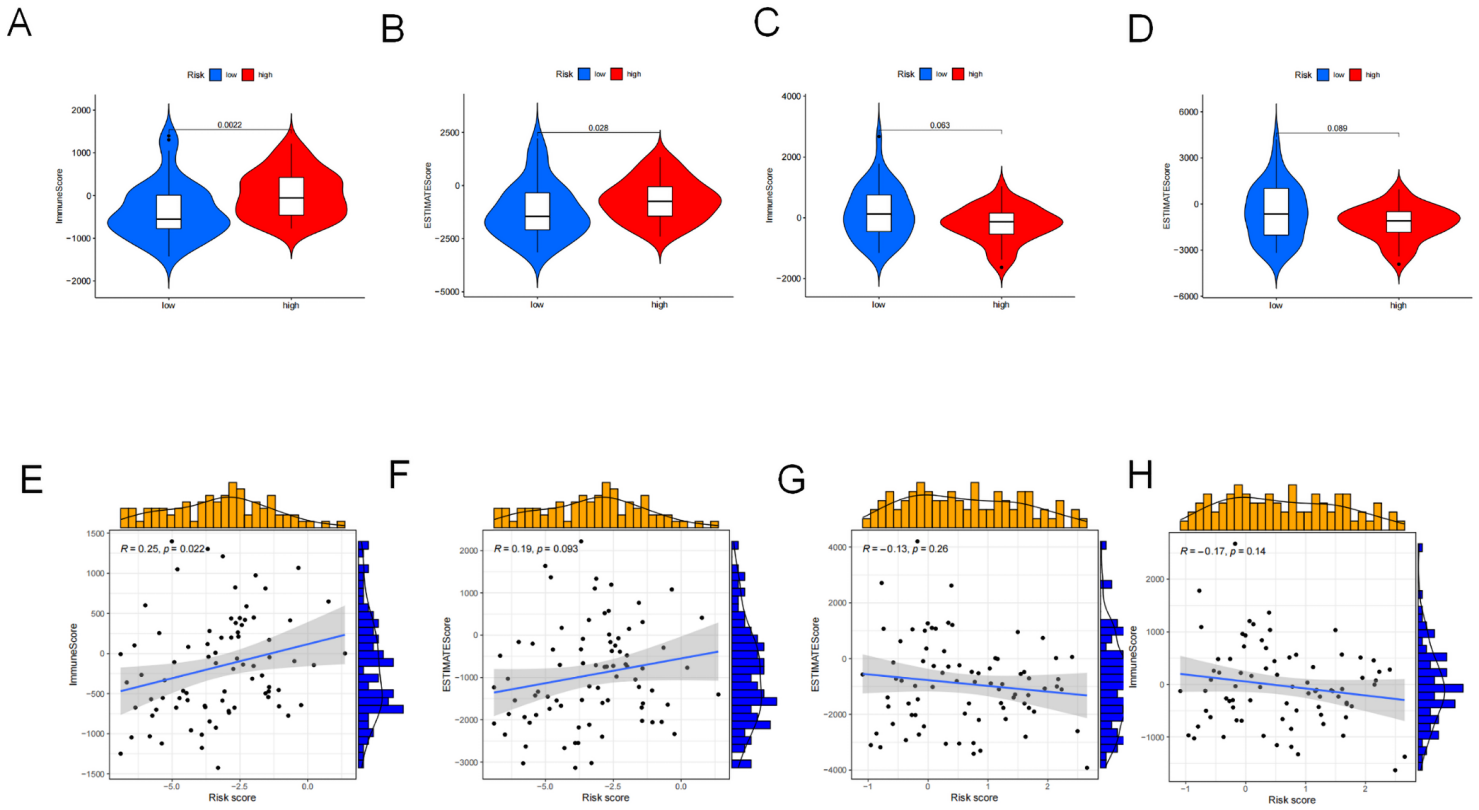
Correlations of immune microenvironments evaluated using ESTIMATE. (A) Immune score and (B) ESTIMATE score for oesophageal squamous cell carcinoma (ESCC). (C) Immune score and (D) ESTIMATE score for oesophageal adenocarcinoma (EAC). Relationships between risk and immune scores for (E) ESCC and (H) EAC. Relationships between risk and ESTIMATE scores for (F) ESCC and (G) EAC.

The ESTIMATE algorithm was used to determine differences between the groups with respect to immune cell infiltration. The high‐risk group of ESCC had higher immune scores than the low‐risk group; similar results were obtained from EAC for the ESTIMATE scores (Figure 7A,B). Meanwhile, the immune cell infiltration and ESTIMATE scores were low in the high‐risk group of EAC (Figure 7C,D). The immune cell infiltration and ESTIMATE scores significantly correlated with risk scores in ESCC (Figure 7E,F) and EAC (Figure 7G,H).

The TIMER2.0 database was used to analyse immune cell infiltration and produce immune cell heatmaps (Figure S4A,B). Most immune cells were positively related to risk scores for ESCC and EAC (Figure S4C,D).

### 3.7 Drug Sensitivity

**FIGURE 8:**
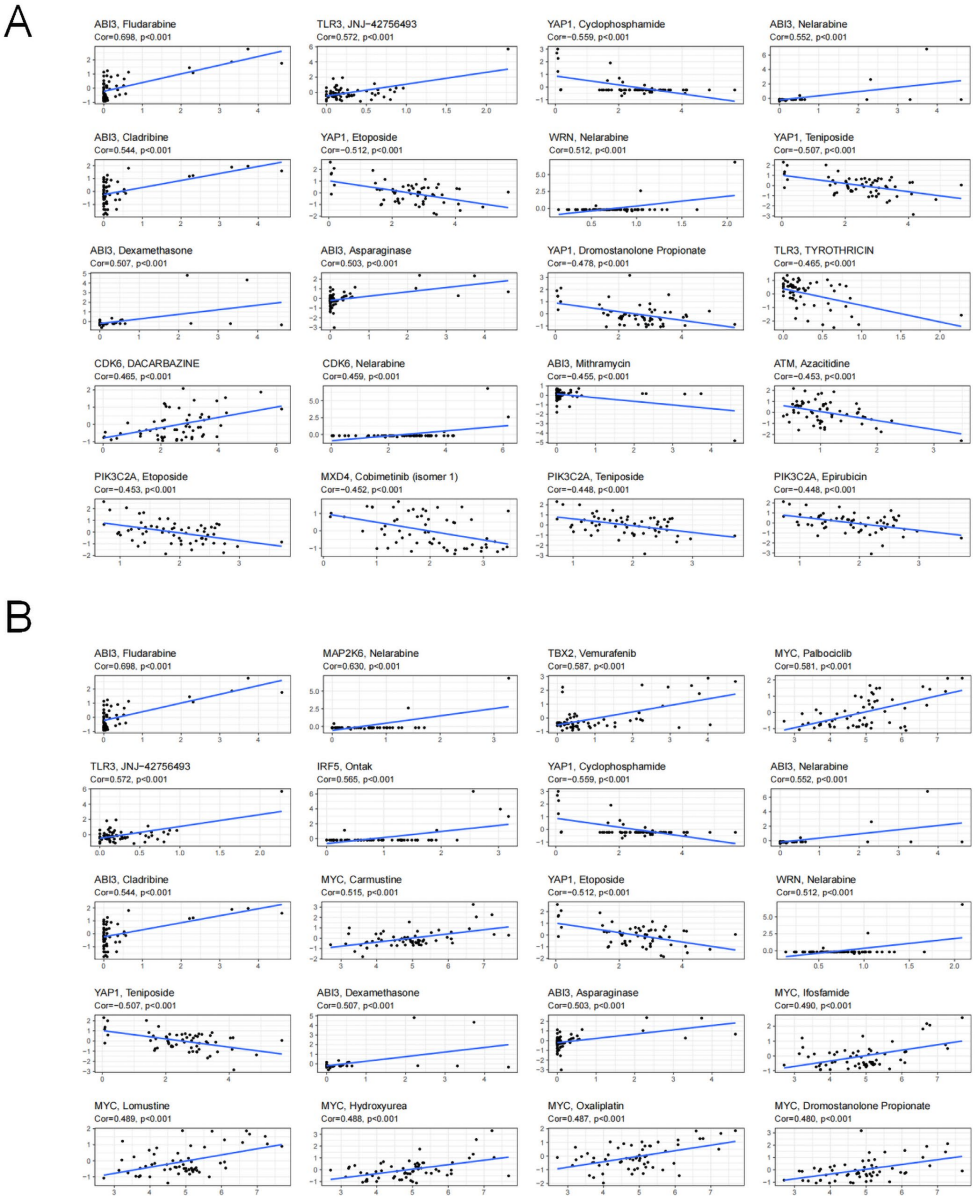
Sensitivity of genes in the signature to chemotherapeutic drugs for (A) oesophageal squamous cell carcinoma and (B) oesophageal adenocarcinoma based on the GDSC database.

**FIGURE 9:**
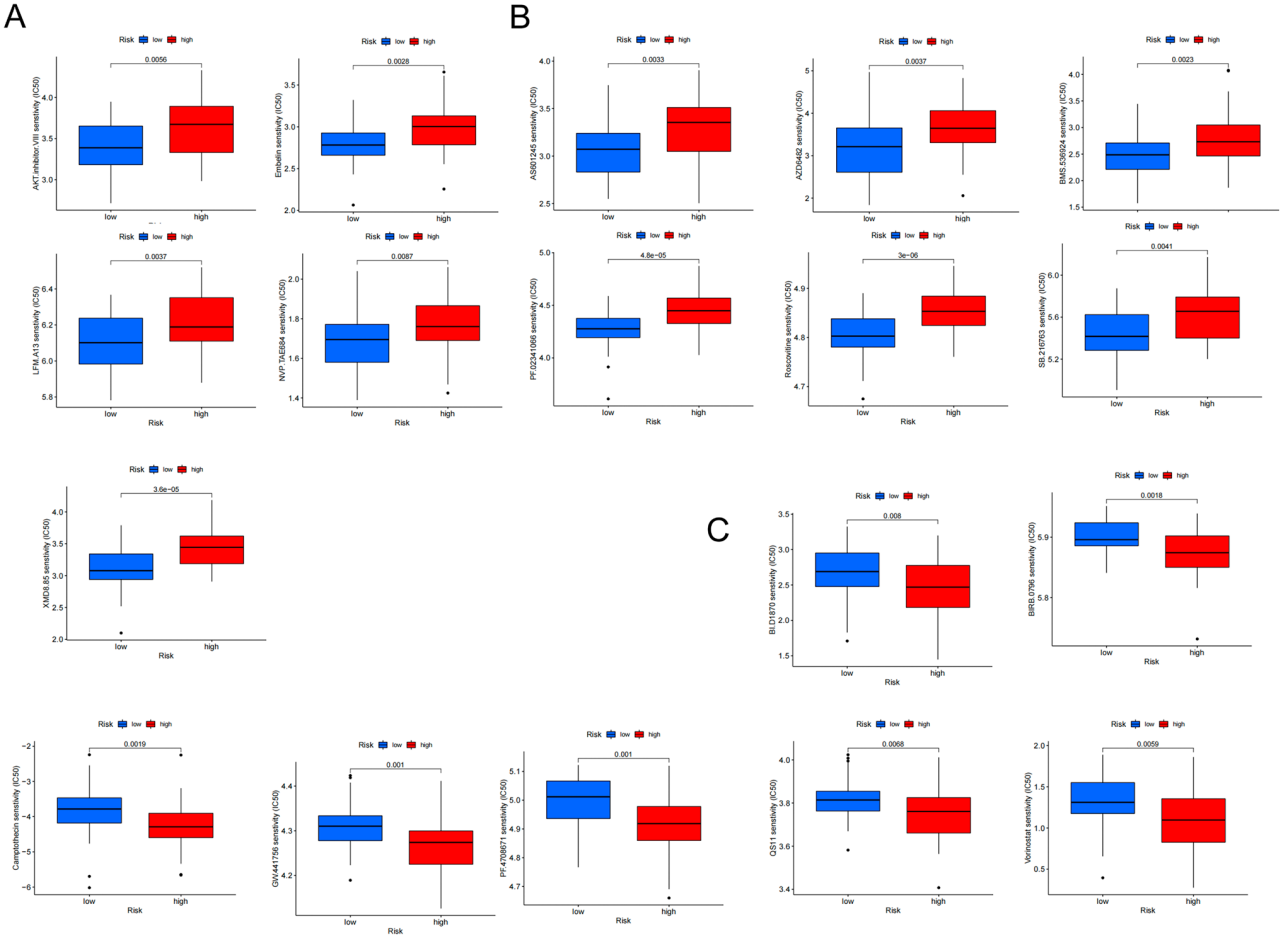
IC_50_ for chemotherapeutic responses of (A) oesophageal squamous cell carcinoma and (B, C) oesophageal adenocarcinoma.

The GDSC database was used to identify candidate drugs that may be useful in treating EC. The data indicated relationships between signature genes and multiple drugs (Figure 8), indicating their potential to guide clinical drug use. There were 18 therapeutic agents with widely varying IC_50_ values. Eleven therapeutic agents had estimated IC_50_ values that indicated increased sensitivity for the high‐risk group, including AKT inhibitor and Embelin for ESCC (Figure 9A), and AS601245, AZD6482, BMS.536924, LFM.A13, NVP.TAE684, PF.02341066, Roscovitine, SB.216763 and XMD8.85 for EAC (Figure 9B). In contrast, the IC_50_ values of BI.D1870, BIRB.0796, camptothecin, GW.441756, PF.4708671, QS11 and vorinostat were low for the high‐risk group compared with those for the low‐risk group (Figure 9C).

### 3.8 Identification of Clinically Relevant Genes

**FIGURE 10:**
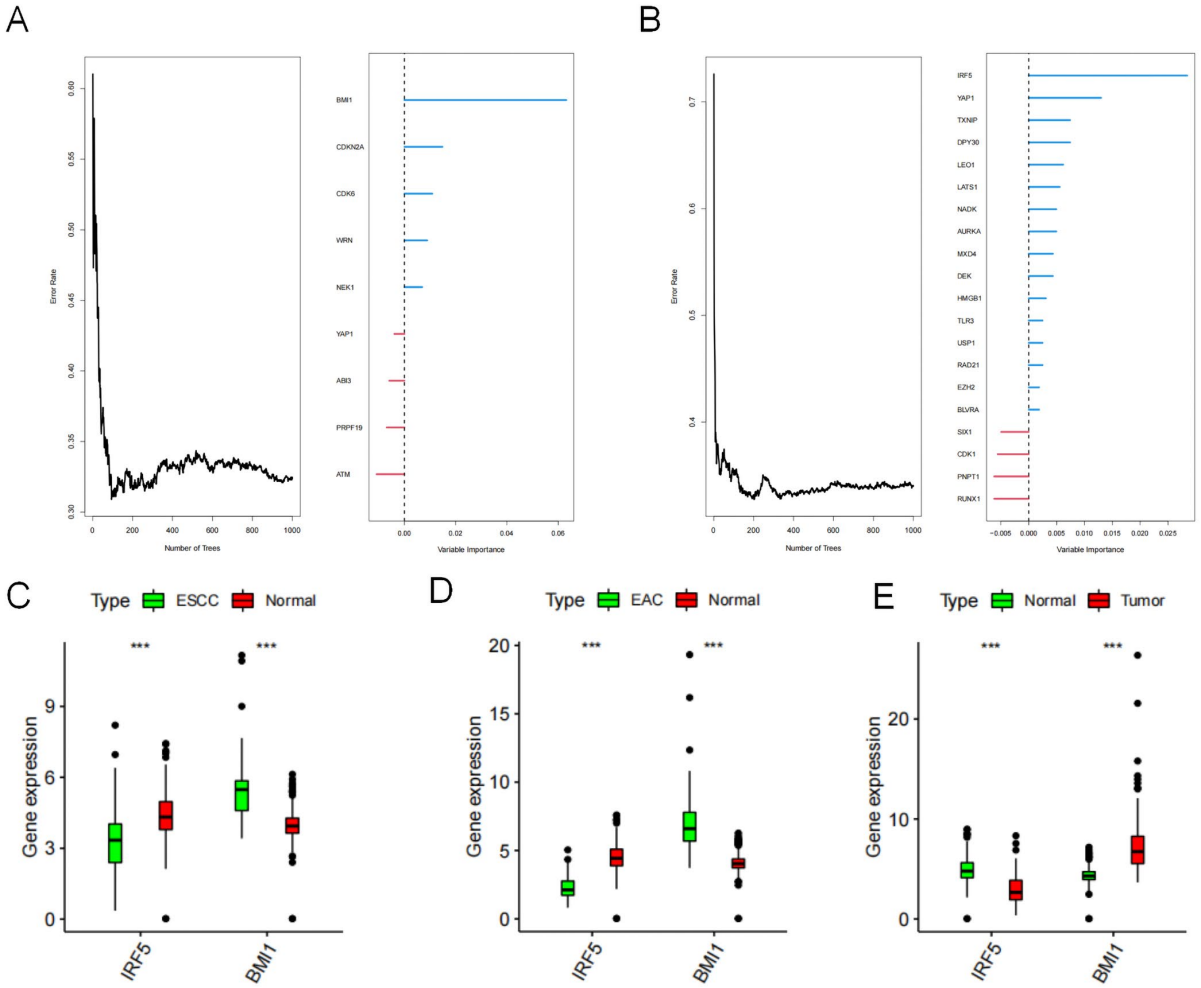
Random forest error rates (left graphs) and relative importance (right graphs) for (A) oesophageal squamous cell carcinoma (ESCC) and (B) oesophageal adenocarcinoma (EAC). Expression of *IRF5* and *BMI1* in (C) ESCC, (D) EAC and (E) EC.

The RSF package was used to identify genes related to high survival significance (relative importance value > 0.5). Figure 10A–C shows the error rates and the numbers of classification trees. *BMI1*, *CDKN2A*, *CDK6*, *WRNN* and *EK1* were the most important genes for ESCC, whereas *IRF5*, *YAP1*, *TXNIP*, *DPY30* and *LEO1* were the most important genes for EAC. As *BMI* and *IRF5* were top on the lists, we selected *BMI* and *IRF5* for further analysis. The expression of these two genes was deconstructed and shown in ESCC (Figure 10C), EAC (Figure 10D) and EC (Figure 10E).

### 3.9 IRF5 Was Over‐Expressed in ESCC Cell Lines, and *IRF5* ‐Knockdown Inhibited the Migration and Proliferation and Promoted the Apoptosis and Senescence of ESCC Cells

**FIGURE 11:**
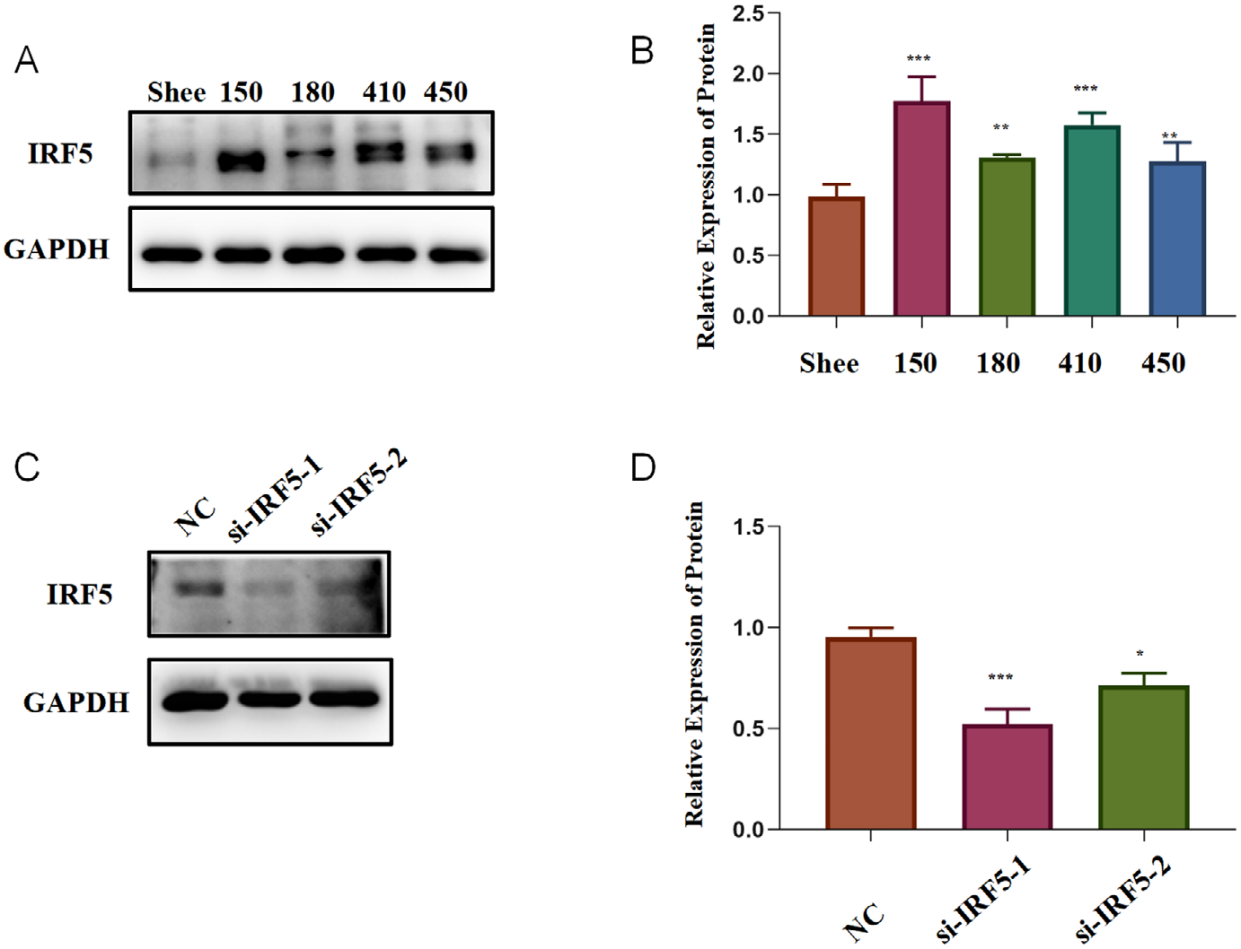
Expression analyses of IRF5 in four ESCC cell lines using western blotting (A, B). The efficiency of *IRF5*‐knockdown in KYSE150 cells was determined using western blotting (C, D). Ctrl: No siRNA infection; NC: Negative control. Statistical analyses of *n* = 3 independent experiments were assessed. Results are shown as mean ± SD, ns *p* ≥ 0.05, *p < 0.05, **p < 0.01, ***p < 0.001.

**FIGURE 12:**
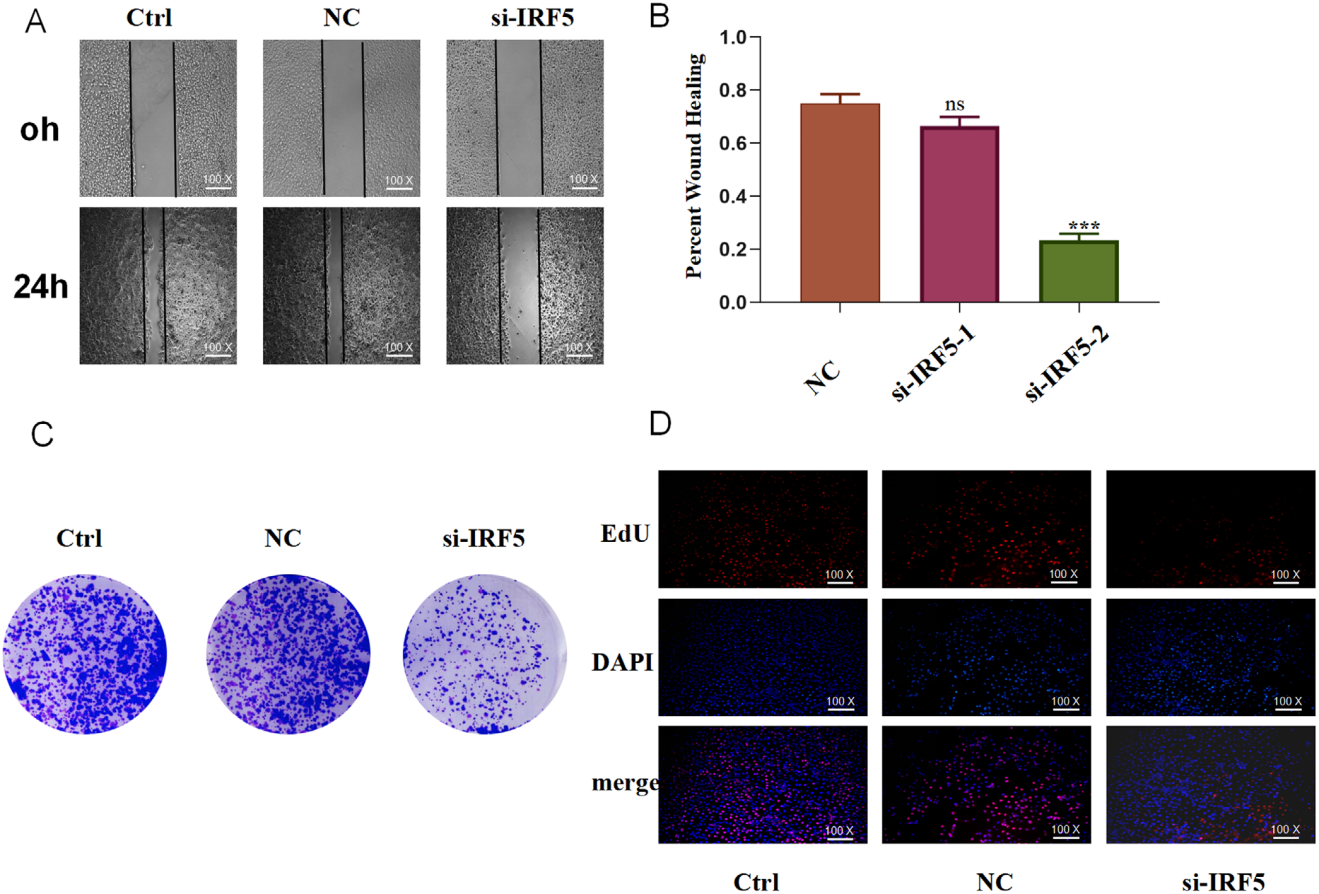
Wound healing assay showing the migration ability of KYSE150 cells (A, B). Clone formation assay (C) and 5‐ethynyl‐2′‐deoxyuridine (EdU) assay (D) were performed to examine the proliferation ability of KYSE150 cells. Statistical analyses of *n* = 3 independent experiments were assessed. Results are shown as mean ± SD, ns *p* ≥ 0.05, ***p < 0.001.

**FIGURE 13:**
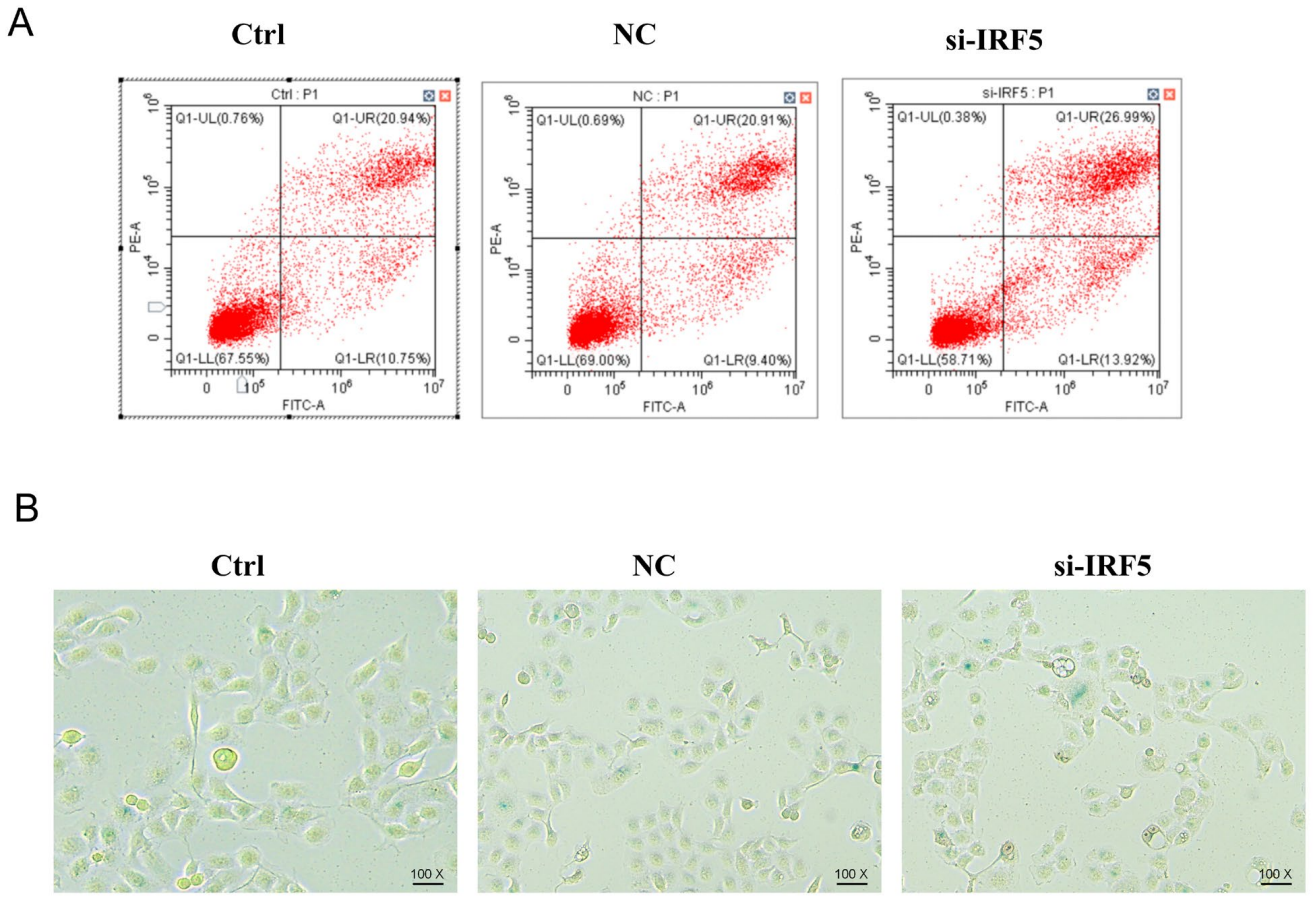
Effect of *IRF5* knockdown on apoptosis of KYSE150 cells was evaluated using an apoptosis assay kit (A). The level of SA‐β‐gal in *IRF5*‐knockdown cells (B). *n* = 3 independent experiments.

We initially determined the level of IRF5 in four ESCC cell lines (KYSE150, KYSE180, KYSE410 and KYSE450) and a normal cell line (Shee) to investigate whether IRF5 plays a role in the biological behaviour of ESCC cells. IRF5 expression was higher in all ESCC cell lines than that in Shee cells (Figure 11A,B). We chose KYSE150 for further analysis since the expression level was the highest and silenced *IRF5* expression using an siRNA. IRF5 expression was considerably reduced (Figure 11C,D) using si‐IRF5‐1, which was then selected for further functional assays. The wound healing assay showed that *IRF5* knockdown led to a significant decrease in cell migration capacity (Figure 12A,B). The colony formation and EdU assays demonstrated that *IRF5* knockdown inhibited the proliferation of ESCC cells (Figure 12C,D). The apoptosis assays showed that *IRF5* knockdown promoted apoptosis in KYSE150 cells (Figure 13A). The percentage of SA‐β‐gal‐positive cells significantly increased in *IRF5*‐knockdown cells (Figure 13B) compared with the control group. Collectively, these results showed that IRF5 is essential for ESCC cell proliferation, migration, apoptosis and senescence.

## 4 Discussion

EC is a common tumour of the digestive system. Locally advanced or unresectable tumours have an extremely poor prognosis, accounting for 50%–60% of all cases [21]. In clinical practice, treatment outcomes vary among patients with tumours of the same pathologic stage and grade in some cases. Although multiple treatments are available, including surgery, radiotherapy, chemotherapy, targeted therapy and immunotherapy [22], individual specificity is not high and the biological characteristics of EC are still being extensively studied to help patients receive the best treatment. In this study, we obtained data from a public database and applied an algorithm for gene pairs. A 19‐SRGP signature for ESCC and a 26‐SRGP signature for EAC were obtained using the univariant Cox and LASSO analyses in this study. These signatures were validated using the K–M and ROC curve analyses. Moreover, our experiments investigating the relationships between two risk groups and immune cell infiltration, together with TME, revealed different immune signatures for the two groups. This finding may help explain their different responses to immune checkpoint inhibitors (ICIs).

The NICE study [23] on a neoadjuvant treatment showed a pathological complete response rate of 39.2% in patients with ESCC receiving ICIs. Meanwhile, the KEYNOTE‐590 study showed that pembrolizumab combined with chemotherapy improved the OS of patients with ESCC, whereas patients with EAC benefited relatively less from the treatment [24]. We also predicted the sensitivity and IC_50_ of chemotherapeutic agents for our two risk groups of patients being treated for EC. We used the random forest method to identify the most clinically relevant genes in these signatures. A gene‐pair comparison strategy does not require data normalisation or consideration of batch effects across platforms, and gene‐pair signatures are generally robust and easy to use.

The most clinically important genes identified using the random forest method were *IRF5* and *BMI1*; we focused on *IRF5*, as *BMI1* was extensively studied in various tumours [25, 26, 27]. *IRF5* encodes interferon regulatory factor 5 (IRF5), an important member of the IRF family. It is primarily expressed in macrophages and can be activated by pathways such as TLR pathways in inflammatory environments [28], further promoting the expression of downstream inflammation‐related genes and mediating the inflammatory response [29, 30, 31]. IRF5 plays an important role in pathogen defence and immune cell differentiation and plays tumour‐suppressive and tumour‐promoting roles in different tumours [32, 33]. Our results showed that IRF5 was overexpressed in ESCC cell lines, and the suppression of IRF5 expression inhibited the migration and proliferation and promoted the apoptosis and senescence of ESCC cells.

Our study has some limitations. It was a retrospective study, and we could not acquire treatment and recurrence records, making the data somewhat incomplete. Also, we could not fully elucidate the biological functions of IRF5 in EC and its mechanisms. Therefore, in vivo and in vitro experiments are required to validate the findings. Our signatures and crucial genes need to be validated in larger randomised controlled trials and experiments to further investigate their role in EC progression and better understand the mechanisms of tumourigenesis. Therefore, our findings should be interpreted with caution.

Overall, we applied a gene‐pair algorithm to analyse SRGs and the TCGA‐EC database with the corresponding clinical features and developed a predictive SRGP signature. Our study showed that patients with EC in different risk groups exhibit diverse immune responses correlating with SRGPs, as well as different sensitivity to therapeutic agents. Moreover, low‐risk patients with EC benefited more from treatment than high‐risk patients. We identified several key prognostic genes that need further studies to validate their roles. This signature is a potential tool to identify high‐risk patients. Moreover, our study provides evidence that *IRF5* may be a promising therapeutic target.

## 5 Conclusion

Our study established the first SRGP signature to predict prognosis in patients with EC. Based on our results, we propose that *IRF5* is a potential target for treating patients with EC.

## Author Contributions

**Long Yao:** formal analysis (lead), writing – original draft (lead). **Xiu Chen:** formal analysis (equal), writing – original draft (equal). **Yanxin Fang:** formal analysis (equal), writing – original draft (equal). **Yunlong Huang:** writing – review and editing (equal). **Kaiming Wu:** validation (equal). **Xin Huang:** supervision (equal). **Junrui Xu:** software (equal). **Renquan Zhang:** conceptualization (lead), supervision (lead).

## Ethics Statement

This study was approved by the Ethical Committee of the First affiliated hospital of Anhui medical university (approval number: PJ2020‐15‐20).

## Consent

As this study involves no more than minimal risk to patients, a waiver of informed consent was requested. All of the procedures were performed in accordance with the Declaration of Helsinki and relevant policies in China, and patients data confidentiality was ensured.

## Conflicts of Interest

The authors declare no conflicts of interest.

## Supporting information


**Figure S1.** Spatial multi‐omics mapping of IRF5 in a patient with liver metastasis from colon cancer. (A) High‐resolution HE‐stained sections. (B) Cellular components based on the deconvolution algorithm. (C) IRF5 spatial transcriptome localisation. (D) Difference in IRF5 expression in the malignant region, mixed malignant region and normal region. (E) The spearman correlation between gene expression and microenvironmental components.

**Figure S2.** Kaplan–Meier analyses of survival of patients with oesophageal cancer stratified by (A, D) tumour size, (B, E) lymph node and (C, F) stage in oesophageal squamous cell carcinoma (A–C) and oesophageal adenocarcinoma (D–F).

**Figure S3.** Differences in biological processes among patients with oesophageal cancer assessed using gene set variation analysis. Kyoto Encyclopedia of Genes and Genomes pathway enrichment for (A) oesophageal squamous cell carcinoma (ESCC) (B) and oesophageal adenocarcinoma (EAC). Hallmark gene sets for (C) ESCC and (D) EAC.

**Figure S4.** Correlations for immune cell infiltration. Heatmaps of immune cell infiltration in (A) oesophageal squamous cell carcinoma (ESCC) and (B) oesophageal adenocarcinoma (EAC). TIMER2.0 immune cell associations for (C) ESCC and (D) EAC.

**Table S1.** LASSO regression coefficients of senescence‐related gene pairs (SRGPs).

## Data Availability

The raw data required to generate the findings are available for download from the UCSC Xena (https://xena.ucsc.edu/).
