# Supplementary figures and images for: Multi‐Omics Analysis of Aberrances and Functional Implications of IRF5 in Digestive Tract Tumours
Source: J Cell Mol Med. 2025 Feb 24;29(4):e70433. doi: 10.1111/jcmm.70433 (PMC11850095; doi:10.1111/jcmm.70433)

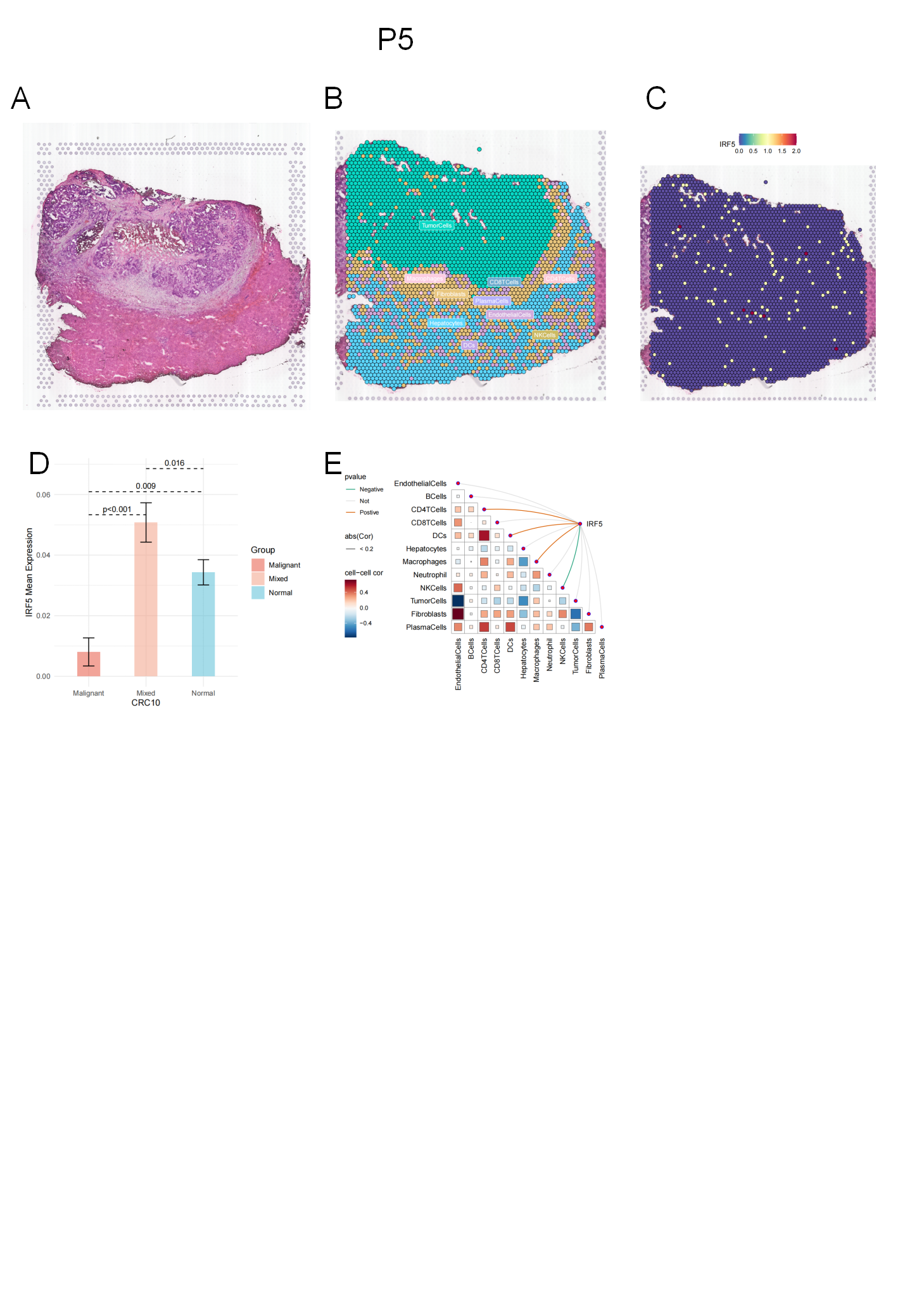

Supplement: Supplementary file 1 — Figure S1. Spatial multi‐omics mapping of IRF5 in a patient with liver metastasis from colon cancer. (A) High‐resolution HE‐stained sections. (B) Cellular components based on the deconvolution algorithm. (C) IRF5 spatial transcriptome localisation. (D) Difference in IRF5 expression in the malignant region, mixed malignant region and normal region. (E) The spearman correlation between gene expression and microenvironmental components. [file JCMM-29-e70433-s002.tif]

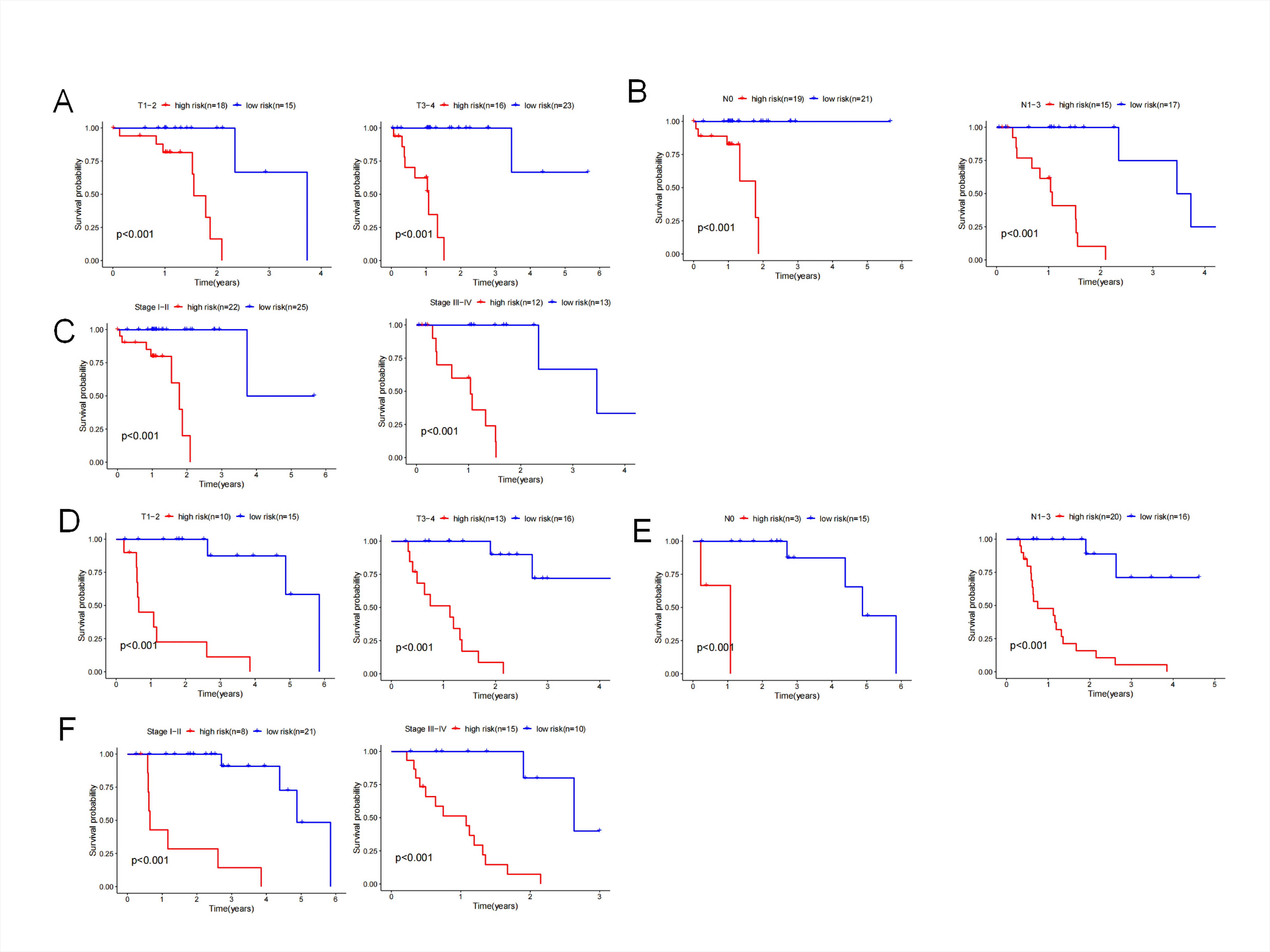

Supplement: Supplementary file 2 — Figure S2. Kaplan–Meier analyses of survival of patients with oesophageal cancer stratified by (A, D) tumour size, (B, E) lymph node and (C, F) stage in oesophageal squamous cell carcinoma (A–C) and oesophageal adenocarcinoma (D–F). [file JCMM-29-e70433-s004.tif]

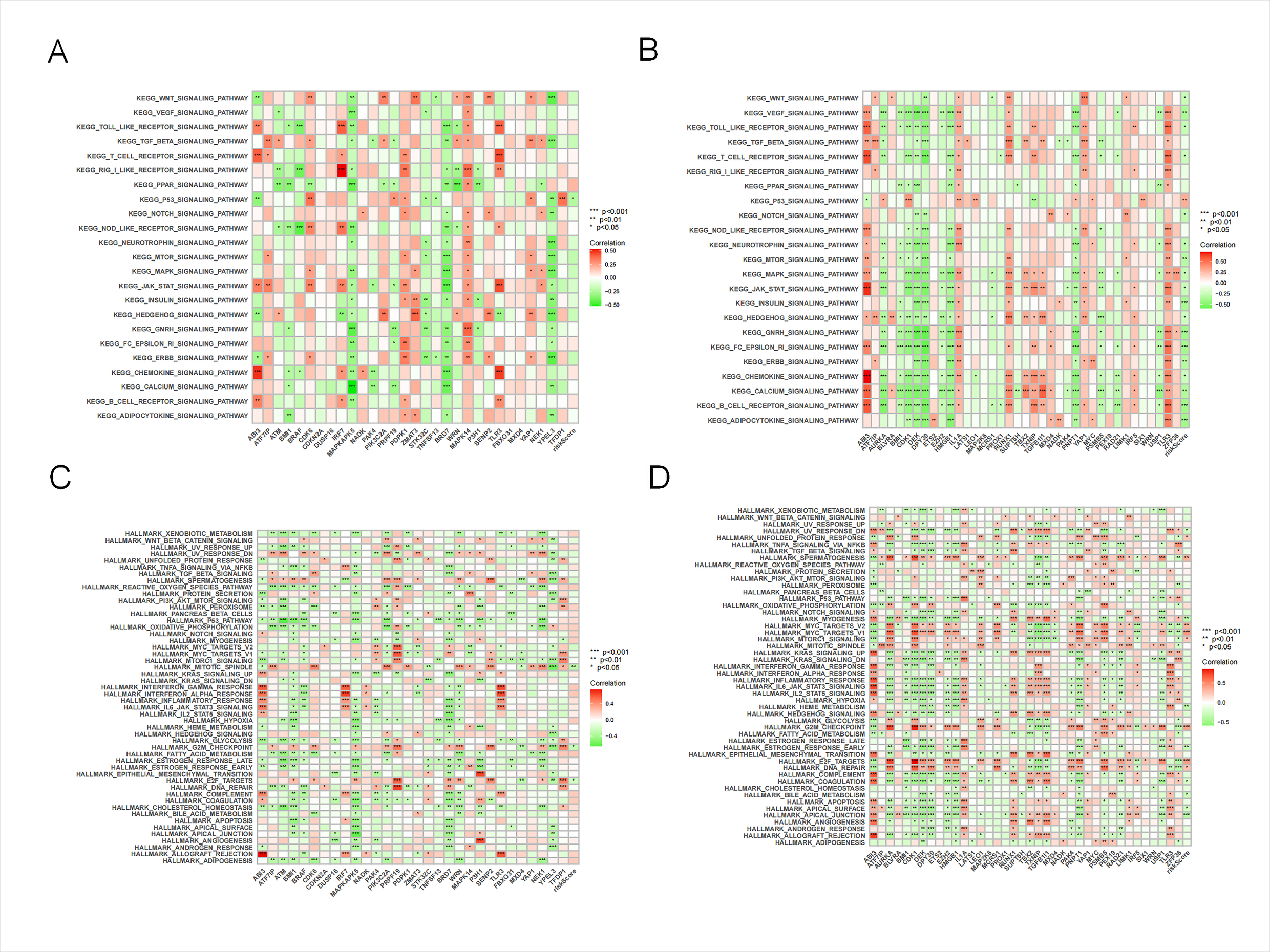

Supplement: Supplementary file 3 — Figure S3. Differences in biological processes among patients with oesophageal cancer assessed using gene set variation analysis. Kyoto Encyclopedia of Genes and Genomes pathway enrichment for (A) oesophageal squamous cell carcinoma (ESCC) (B) and oesophageal adenocarcinoma (EAC). Hallmark gene sets for (C) ESCC and (D) EAC. [file JCMM-29-e70433-s003.tif]

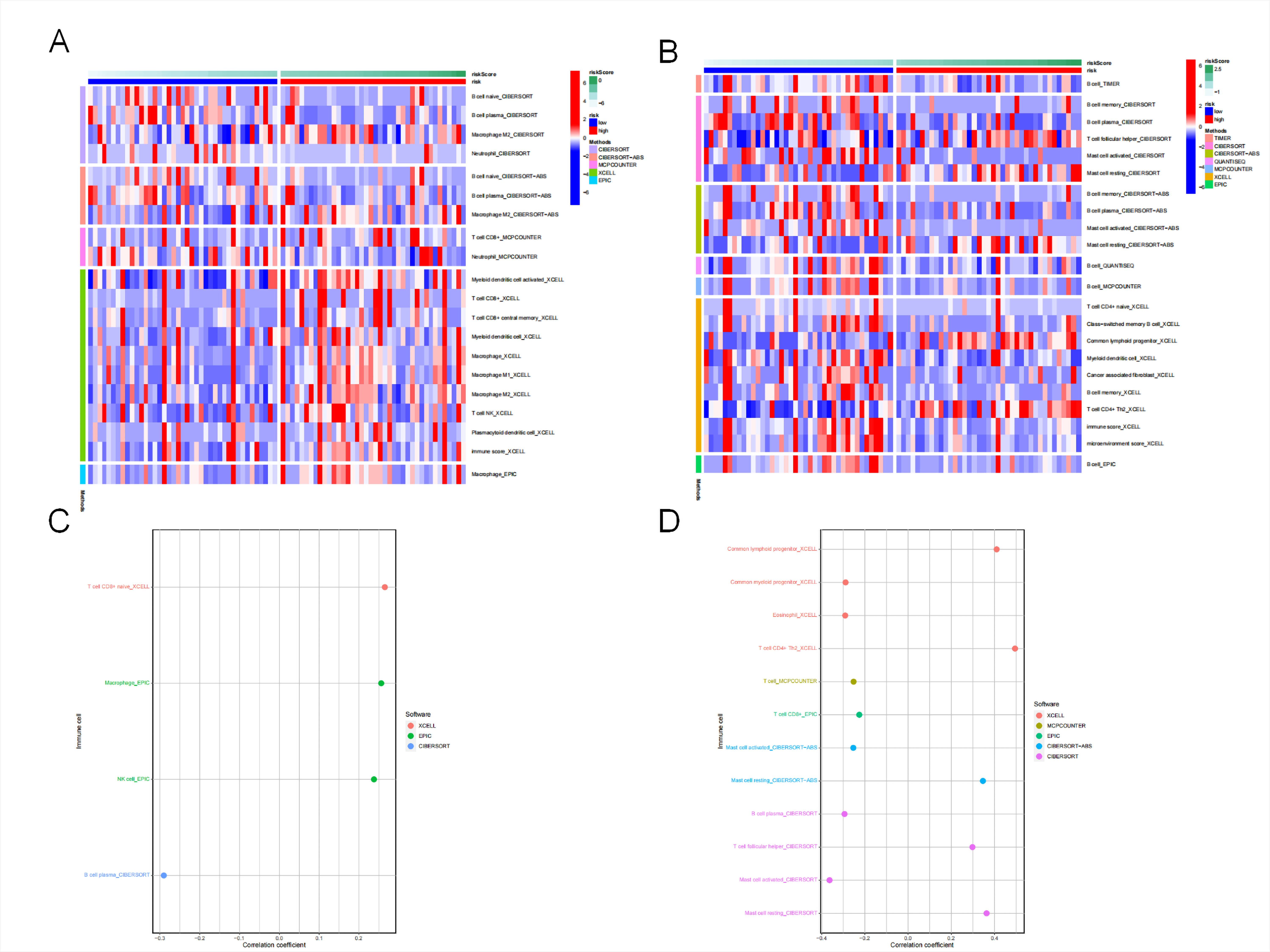

Supplement: Supplementary file 4 — Figure S4. Correlations for immune cell infiltration. Heatmaps of immune cell infiltration in (A) oesophageal squamous cell carcinoma (ESCC) and (B) oesophageal adenocarcinoma (EAC). TIMER2.0 immune cell associations for (C) ESCC and (D) EAC. [file JCMM-29-e70433-s001.tif]
