# Supplementary material for: Multi‐Omics Analysis of Aberrances and Functional Implications of IRF5 in Digestive Tract Tumours
Source: J Cell Mol Med. 2025 Feb 24;29(4):e70433. doi: 10.1111/jcmm.70433 (PMC11850095; doi:10.1111/jcmm.70433)
Supplement: Supplementary file 5 — Table S1. LASSO regression coefficients of senescence‐related gene pairs (SRGPs). [file JCMM-29-e70433-s005.docx]

| Gene pairs |  | Coefficient |  |
| --- | --- | --- | --- |
| ESCC |  |  |  |
| ABI3\|PDPK1 |  | 0.041725609 |  |
| ABI3\|ZMAT3 |  | 0.043082313 |  |
| ATF7IP\|CDK6 |  | 0.103301954 |  |
| ATM\|STK32C |  | -0.50977355 |  |
| ATM\|TNFSF13 |  | -0.294090061 |  |
| BMI1\|BRD7 |  | -1.213856289 |  |
| BRAF\|WRN |  | 0.324530711 |  |
| CDK6\|MAPK14 |  | -0.567375962 |  |
| CDK6\|P3H1 |  | -0.505376905 |  |
| CDKN2A\|SENP2 |  | -0.442682115 |  |
| CDKN2A\|TLR3 |  | -0.324214462 |  |
| DUSP16\|FBXO31 |  | -0.72862774 |  |
| DUSP16\|MXD4 |  | -0.022787278 |  |
| IRF7\|YAP1 |  | 0.943736596 |  |
| MAPKAPK5\|NEK1 |  | 0.401527345 |  |
| NADK\|PAK4 |  | 0.722682028 |  |
| PAK4\|YPEL3 |  | -0.459686046 |  |
| PIK3C2A\|SENP2 |  | -0.099462716 |  |
| EAC |  |  |  |
| ABI3\|TGFB1I1 |  | 0.28240582 |  |
| ATF7IP\|MXD4 |  | 0.066955512 |  |
| AURKA\|NADK |  | 0.033911498 |  |
| AURKA\|PAK4 |  | 0.247609155 |  |
| BLVRA\|EZH2 |  | -0.028915057 |  |
| BLVRA\|LATS1 |  | -0.334638687 |  |
| BLVRA\|PNPT1 |  | -0.044830994 |  |
| BMI1\|YAP1 |  | 0.123880059 |  |
| CDK1\|NADK |  | 0.133070963 |  |
| DEK\|MYC |  | 0.047516816 |  |
| DEK\|PSMB5 |  | 0.224342658 |  |
| DPY30\|PEX19 |  | 0.799941219 |  |
| ETS2\|RAD21 |  | -0.028605064 |  |
| EZH2\|LIMK1 |  | 0.17284316 |  |
| HMGB1\|PSMB5 |  | 0.029375 |  |
| HMGB1\|YAP1 |  | 0.164920485 |  |
| IL1A\|IRF5 |  | 0.207595986 |  |
| LATS1\|LIMK1 |  | 0.140776689 |  |
| LEO1\|NADK |  | 0.058720311 |  |
| MAP2K6\|SIX1 |  | -0.359318755 |  |
| MCRS1\|YAP1 |  | 0.006309421 |  |
| PROX1\|SIX1 |  | -0.103259934 |  |
| RUNX1\|WRN |  | -0.036777512 |  |
| SUPT5H\|USP1 |  | -0.355465791 |  |
| TBX2\|TLR3 |  | 0.58266077 |  |
| TXNIP\|ZFP36 |  | -0.06645287 |  |

Table S1 Lasso regression coefficients of SRGPs
